# Test for Respiratory and Asthma Control in Kids (TRACK): validation of the Portuguese version

**DOI:** 10.1186/s40413-018-0219-y

**Published:** 2018-12-05

**Authors:** Gustavo F. Wandalsen, Renata G. Dias, Herberto J. Chong-Neto, Nelson Rosário, Lillian Moraes, Neusa F. Wandalsen, Décio Medeiros, Ana Caroline Della Bianca, Marilyn Urrutia-Pereira, Jennifer Avila, Patrícia P. Jorge, Dirceu Solé

**Affiliations:** 1 Federal University of São Paulo (UNIFESP), Rua dos Otonis 725, São Paulo, SP 04025-002 Brazil; 2 Federal University of Paraná (UFPR), Curitiba, Brazil; 3 Federal University of Mato Grosso (UFMT), Cuiabá, Brazil; 4 ABC Medical School, Santo André, Brazil; 5 Federal University of Pernambuco (UFPE), Recife, Brazil; 6 Federal University of Pampas (UNIPAMPA), Bagé, Brazil; 7 Federal University of São Carlos (UFSCAR), São Carlos, Brazil

**Keywords:** Asthma, Wheezing, Children, Control

## Abstract

**Background:**

TRACK (Test for Respiratory and Asthma Control in Kids) questionnaire is an instrument developed and validated in English to evaluate the control of respiratory symptoms in children under 5 years of age.

**Objective:**

To validate the Portuguese version of the TRACK questionnaire.

**Methods:**

The validation was done in an observational, prospective and multicenter evaluation (six centers in Brazil) in children with recurrent respiratory symptoms. Children were classified according to symptoms, GINA criteria and medical evaluation. Parents and doctors rated child respiratory symptom control in the last month (VAS). Approval from the Institutional Review Board was obtained in each centre, and written informed consent was obtained from parents.

**Results:**

Data from 299 children were obtained at baseline, and 195 at follow-up. The median score of the TRACK questionnaire was 65 and Cronbach’s α was 0.70. TRACK scores showed significant correlation with the medical and family opinions about symptom control (r: 0.74 and r: 0.61). TRACK scores were significantly lower in children who had used systemic steroids (median [IQR]: 45 [30–65] vs 75 [55–80]; *p* < 0.001) and had an emergency visit in the last month (45 [35–60] vs 70 [55–80]; *p* < 0.001). TRACK scores were also significantly different when children were separated by the medical opinion, GINA criteria and symptoms. Comparison of different respiratory symptom control cut-off points showed that the cut-off of 80 points had the highest area under ROC curve (0.800).

**Conclusion:**

We have demonstrated that the Portuguese version of the TRACK questionnaire has satisfactory reliability (internal consistency), adequate criterion validity (compared against GINA levels of control) and constructive validity (compared against respiratory symptoms and medical opinion), showing that it can be a useful tool to discriminate among children with different levels of respiratory symptom control.

**Trial registration:**

ClinicalTrials.gov: NCT03290222.

## Background

Asthma is the most prevalent chronic disease in childhood, and many patients have symptoms early in life [1]. At this age, asthma management is particularly challenging, with greater difficulty in diagnosis confirmation, limitation of additional tests (such as lung function) and available control medication.

In Brazil, asthma is highly prevalent among children, affecting approximately 1 in 4  school children and 1 in 5 teenagers [2]. A recent study demonstrated that about 50% of infants in Brazil presented at least one episode of wheezing in the first year of life and almost 25% had recurrent wheezing [3].

Recent international consensus emphasizes the importance of asthma management by level of control [4], and various instruments have been developed to support this classification, especially written questionnaires for assessment of asthma control. These questionnaires, such as the Asthma Control Questionnaire (ACQ) and the Asthma Control Test (ACT) were initially developed for adults and subsequently adapted for children, like the Childhood Asthma Control Test (C-ACT) [5–7].

Until now, the TRACK (Test for Respiratory and Asthma Control in Kids) questionnaire is the only instrument developed and validated to evaluate the control of respiratory symptoms in young children under 5 years of age [8]. The TRACK questionnaire consists of 5 questions, scored 0 to 20 points, to be answered by caregivers of children with asthma or recurrent respiratory symptoms, regarding symptoms, nocturnal awakenings and limitation of daily activities in the last 4 weeks, use of bronchodilators in the last 3 months and systemic corticosteroids in the last year [8].

In the original validation studies conducted in the USA, it was shown that the questionnaire is a valid and sensitive instrument for identifying preschool children with difficult control of respiratory symptoms [8, 9]. The cutoff score of 80 points was identified as the most suitable to discriminate between children with symptom control from those with uncontrolled symptoms, with high sensitivity (83%) and specificity (78%) when compared to consensus criteria [8]. It was also identified that changes of 10 or more points would be clinically relevant and indicated changes in symptom control [9].

The TRACK questionnaire was originally developed in English, and the it has been translated to and validated in Spanish [10] and Arabic [11, 12]. Until now, there is no validation of the TRACK questionnaire translated into Portuguese.

## Methods

The translation of the TRACK questionnaire into Portuguese (Brazilian culture) was done by professional translators. After receiving the Portuguese version of the TRACK questionnaire, it was applied to parents of patients with asthma and/or respiratory symptoms to evaluate comprehension of the questionnaire. The cognitive testing was a cross-sectional, observational, qualitative evaluation with face-to-face interviews of a convenience sample of 10 children and their parents/caregivers. The authors considered that the comprehension of the questionnaire questions was satisfactory, and no changes in the questions would be necessary.

The validation of the Portuguese version of the TRACK questionnaire was done in an observational, prospective, longitudinal and multicenter evaluation. Children with recurrent respiratory symptoms attended at pediatric clinics were included in the study if they met the following inclusion criteria: age up to 5 years; medical diagnosis of asthma or history of at least 3 episodes of cough and/or wheeze and/or dyspnea (duration longer than 24 h) and treated with bronchodilators; native Portuguese speaking families. Exclusion criteria were: other relevant pulmonary diseases (i.e., cystic fibrosis, bronchopulmonary dysplasia); systemic diseases (i.e., heart diseases, neurologic disorders); congenital malformations; illiteracy and/or inability of parents to understand the questionnaire.

There was only 1 follow-up visit 4 weeks (3 to 5 weeks) after the inclusion in the study. Parents completed the TRACK questionnaire in both visits and answered additional questions about respiratory symptoms and use of medication. Children were classified as controlled, partly controlled and uncontrolled, according to Global Initiative for Asthma (GINA) [4] criteria. Medical evaluation was carried out by the attending allergy specialists, who were blinded to the questionnaires’ answers. Children's symptom control was also classified in controlled, partly controlled and uncontrolled based on the clinical judgment of the physicians. Patients were further divided according to their symptoms, employing criteria used in the original validation of the TRACK questionnaire, in symptomatic (respiratory symptoms in the last 4 weeks), with recent symptoms (symptoms in the last year, but not in the last 4 weeks) and asymptomatic (without respiratory symptoms in the last year) [8]. Parents and doctors rated child respiratory symptom control in the last 4 weeks by means of 100 mm visual analog scale (VAS). Demographic and clinical data were obtained from medical records. Vital signs were checked in both visits. Weight (kg) and height (cm) were obtained only at first visit.

According to previous sample size calculation, at least 200 completed questionnaires at first assessment and 106 in longitudinal evaluation (53 of clinically stable group and 53 of unstable group) were necessary [10]. Thus, considering possible losses, we intended to include at least 220 children, with longitudinal evaluation in at least 140. Eligible children in each centre were screened for the study in sequential order until the estimated number of included children was completed.

The statistical analysis was carried out using SPSS 18.0 (SPSS Inc., Chicago, IL, USA). The level of significance was set at *p* < 0.05. The population characteristics were described using standard statistical techniques. Non parametric tests were used since data did not show normal distribution. The TRACK internal consistency was studied by the Cronbach’s α test. The TRACK criterion validation was performed by the comparison of the scores obtained from patients in the three levels of control, according to GINA criteria with Kruskal-Wallis one-way analysis of variance. Constructive validation was done by comparing the TRACK notes between patients discriminated according to their respiratory symptoms (symptomatic, recent symptoms, and asymptomatic) and to the medical opinion with Kruskal-Wallis one-way analysis of variance. Correlation between parent’s and medical VAS symptom control, and TRACK were calculated by Spearman’s coefficient test. Guyatt’s responsiveness index (GRI) was calculated by dividing the mean change of TRACK scores in the unstable group by the standard deviation (SD) of change in the stable group [13]. The test-retest reliability was assessed using intra-class correlation coefficient in clinically stable patients between visits.

Receiver operating characteristic (ROC) curves were constructed to establish TRACK cutoff score values, according to the GINA criteria of respiratory symptom control (controlled vs. partially controlled and uncontrolled symptoms). The minimal clinically important difference (MCID) for TRACK, which is the smallest difference that parents perceive as either beneficial or harmful, was established using anchor-based (GINA and medical classification of respiratory symptoms control, emergency visit due to wheezing in the last month) and distribution-based (0.5 SD and 1 standard error of the mean [SEM] at baseline and at follow-up) methods [9].

The study was initiated in each centre after approval by the local Institutional Review Board and after approval by the coordinating centre. Prior to participation in the study, written informed consent was obtained from parents.

## Results

A total of 312 children were included, but 13 were excluded due to inconsistencies or incomplete data. One hundred and ninety-eight children returned for the follow-up visit in the pre-established time interval and complete data could be obtained in 195 of these children. A total of 299 questionnaires were correctly answered (60 from São Paulo-SP, 50 from Curitiba-PR, 50 from Santo André-SP, 50 from Cuiabá-MT, 45 from Recife-PE and 44 from Uruguaiana-RS) with a predominance of males (61%). Demographical data are shown in Table 1 and clinical data in Table 2.

**Table 1:** Demographical data from infants and preschool children (*N* = 299)

Characteristic	
Male gender	183 (61%)
Age (years)^a^	2.0 (1.0–3.0)
Height (cm)^a^	93 (82–101)
Weight (kg)^a^	14.0 (11.5–17.0)
Premature delivery	52 (17%)

^a^median and interquartile range (IQR)

**Table 2:** Clinical data from infants and preschool children (*N* = 299)

Characteristic	*N* (%)
Wheezing episode in the last 12 months	282 (94)
Age of the first wheeze (months)^a^	6 (1–12)
Hospitalization in the last 12 months	147 (49)
Use of systemic steroid in the last 12 months	261 (45)
Pneumonia in the last 12 months	134 (45)
ED visit in the last month	99 (33)
Wheezing in the last month	150 (50)
Use of systemic steroid in the last month	107 (36)
GINA classification	
Well controlled	85 (28)
Partially controlled	109 (37)
Not controlled	105 (35)
Medical opinion about symptom control	
Well controlled	114 (38)
Partially controlled	103 (35)
Not controlled	82 (27)

^a^median and interquartile range

**Fig. 1:**
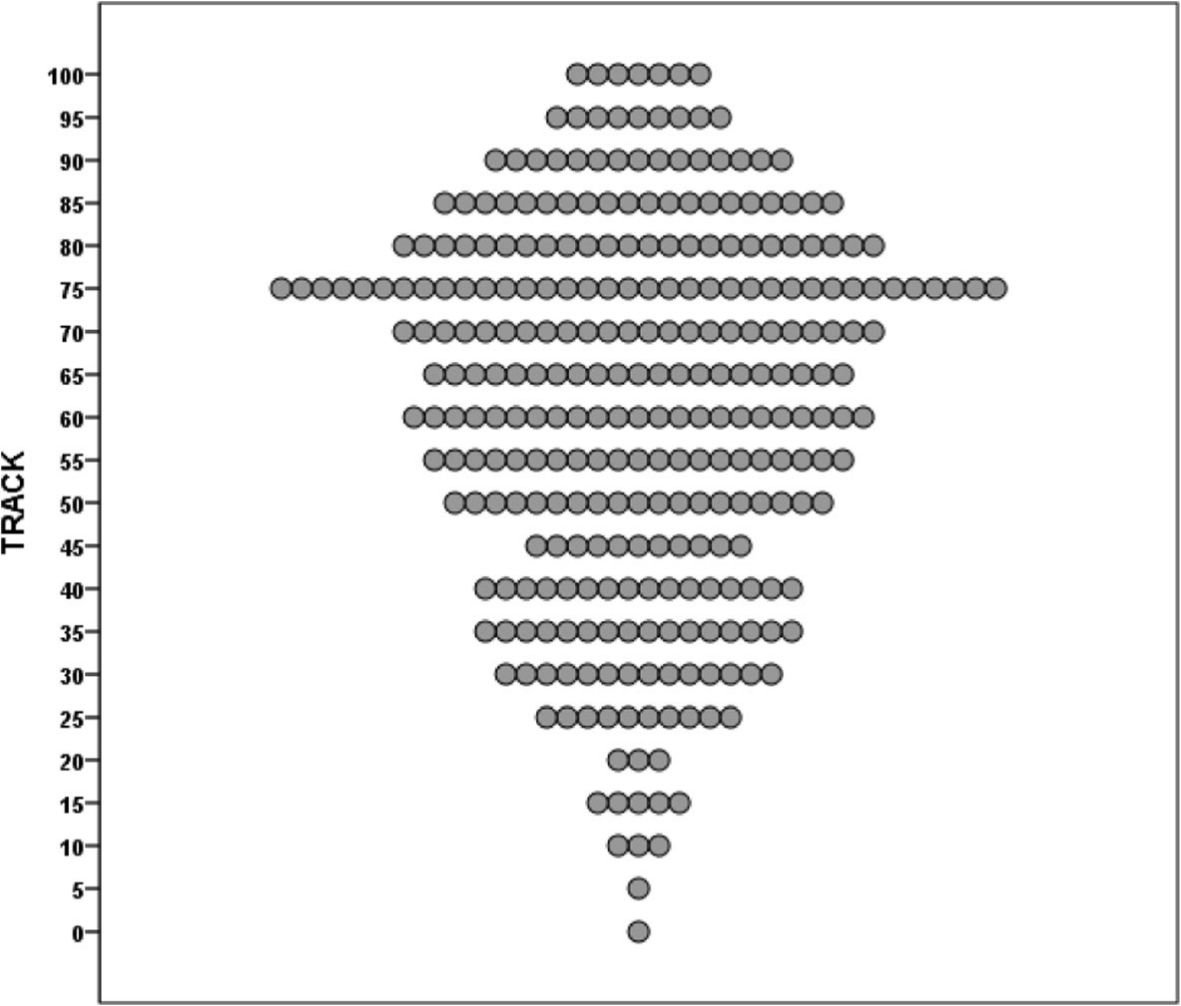
Individual scores of the TRACK questionnaire (*N* = 299)

The median score of the TRACK questionnaire was 65 (IQR: 45–80), ranging from 0 to 100 points and individual scores are shown in Fig. 1. Scores below 80 points were observed in 224 (75%) children. Internal consistency of the questionnaire was acceptable (Cronbach’s α of 0.70).

**Fig. 2:**
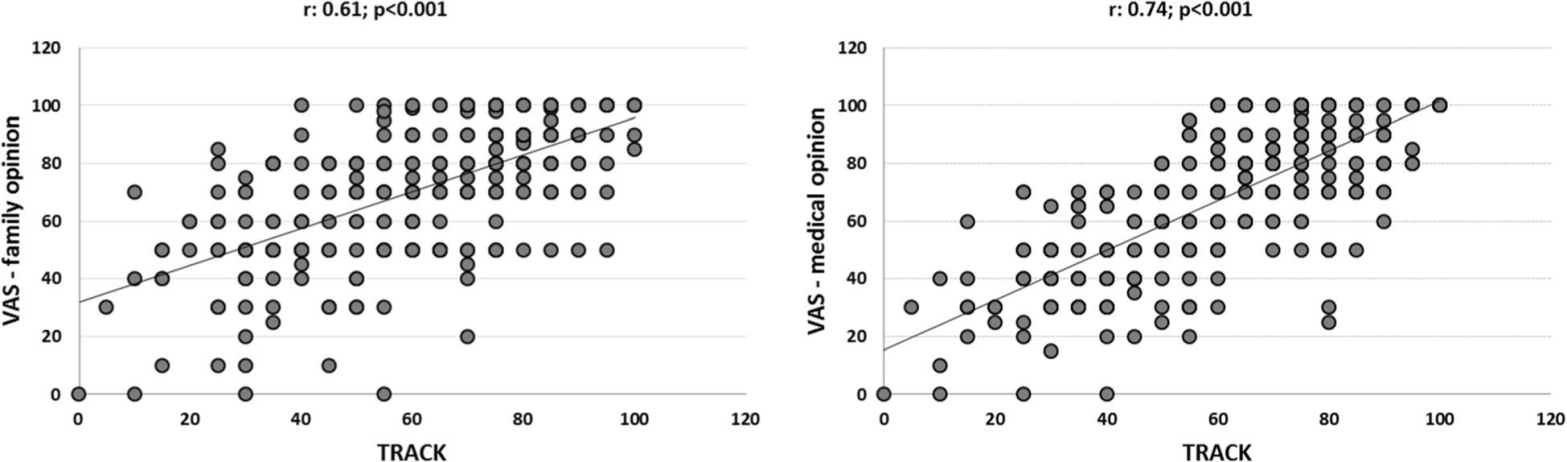
Correlation between TRACK scores and VAS family and medical opinions about symptom control (*N* = 299)

TRACK scores showed strong correlation with the medical opinion (VAS) about symptom control (r: 0.74; *p* < 0.001) and moderate correlation with the family opinion (r: 0.61; *p* < 0.001), as shown in Fig. 2.

**Fig. 3:**
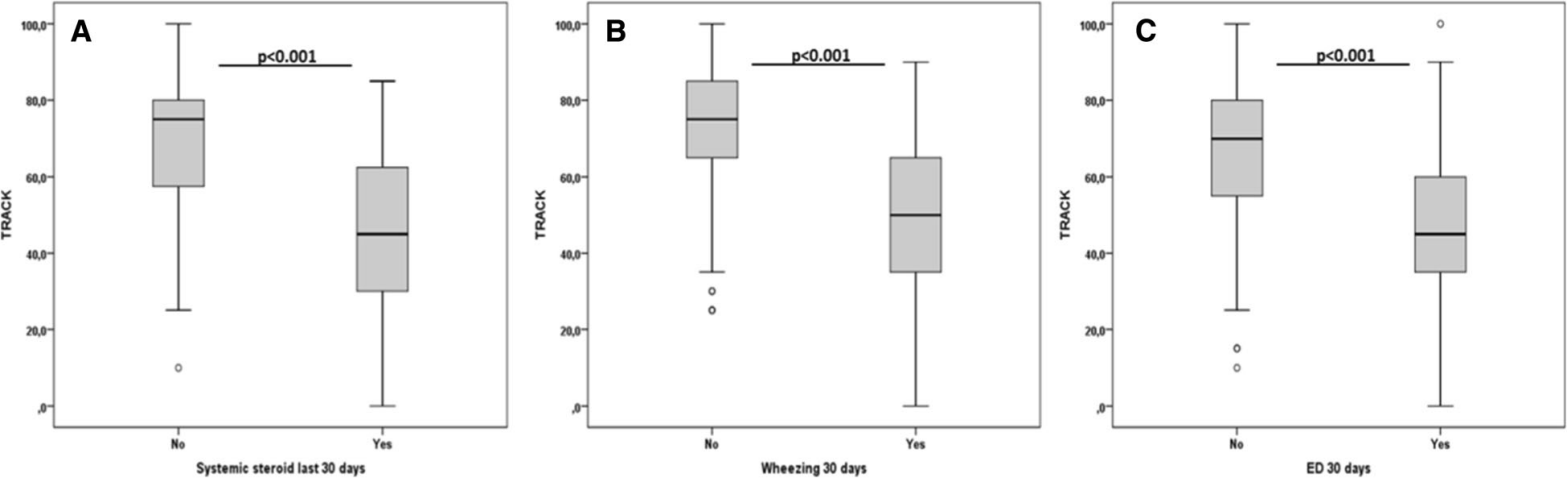
TRACK scores according to the use of systemic steroids in the last month (**a**), wheezing in the last month (**b**) and an emergency visit in the last month (**c**)

**Fig. 4:**
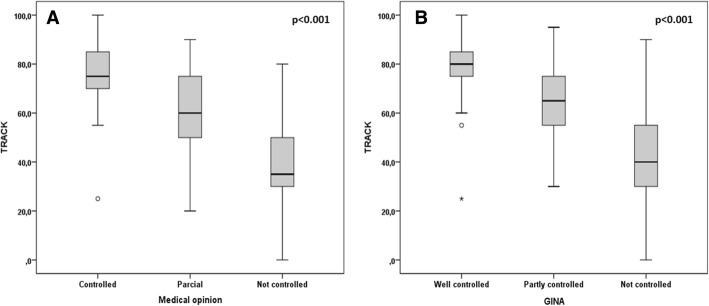
TRACK scores according to medical opinion about symptom control (**a**) and to GINA criteria of symptom control (**b**)

TRACK scores were significantly lower in children who had used systemic steroids in the last month (median [IQR]: 45 [30–65] vs 75 [55–80]; *p* < 0.001), wheezed in the last month (50 [35–65] vs 75 [60–85]; *p* < 0.001) and had an emergency visit in the last month (45 [35–60] vs 70 [55–80]; *p* < 0.001) (Fig. 3). TRACK scores were also significantly different when children were separated by the medical opinion about symptom control and by GINA criteria of symptom control (Fig. 4).

When preschool children were analyzed by symptoms, 6% were asymptomatic (no symptoms in the last year), 44% were recently symptomatic (symptoms in the last year, but not in the last month), and 50% were symptomatic (symptoms in the last month). TRACK scores were significantly different among these groups (85 [75–95] vs 75 [60–85] vs 50 [35–65], respectively; *p* < 0.001).

Comparison of different respiratory symptom control cut-off points of TRACK questionnaire is shown in Table 3 and the cut-off of 80 points has had higher area under ROC curve (AUC; 0.800).

**Table 3:** TRACK cut-off points to separate children with and without respiratory symptom control

Cut-off	AUC^a^	Sensitivity (%)	Specificity (%)
< 70 points	0.793	64.4	94.1
< 75 points	0.785	71.0	85.9
< 80 points	0.800	79.9	80.0
< 85 points	0.703	86.4	54.1
< 90 points	0.652	91.6	38.8

^a^area under ROC curve

Mean difference in TRACK scores according to several anchor-based and distribution-based approach to MCID ranged from 9.3 points to 24.6 (Table 4).

**Table 4:** Minimal clinical important differences (MCID) of TRACK according to anchor-based and distribution-based approaches

	Mean difference in TRACK scores	Effect size
Medical opinion		
Well vs partially controlled	15.6	0.69
Partially vs not controlled	24.6	1.1
GINA classification		
Well vs partially controlled	15.7	0.69
Partially vs not controlled	20.6	0.91
ED visit in the last month		
Yes vs no	20.7	0.92
Medical opinion at follow-up (*N* = 195)		
Better/worse (1 level)	9.3	0.41
Baseline (*N* = 299) – 0.5 SD	11.3	–
Follow-up (*N* = 195) – 0.5 SD	10.7	–
Baseline (*N* = 299) – 1 SEM	12.4	–
Follow-up (*N* = 195) – 1 SEM	11.7	–

*ED* emergency department; *SD* standard deviation; *SEM* standard error of the mean

One hundred ninety five children returned for follow-up visit and had questionnaires correctly answered. From these, 83 (43%) were clinically stable (no change in GINA classification) and 112 (57%) were unstable. The difference in TRACK scores between visits from these two groups of children was significantly different (∆: 5 [5–15] vs 20 [11–25); *p* < 0.001). The intra-class correlation coefficient of the TRACK measurements among clinically stable children was 0.85 (95% CI: 0.77–0.91) and Guyatt’s responsiveness index was 1.54.

## Discussion

The results of our study clearly show that the Portuguese version of the TRACK questionnaire has adequate psychometric proprieties. We have demonstrated satisfactory reliability (internal consistency), adequate criterion validity (compared against GINA levels of control) and constructive validity (compared against respiratory symptoms and medical opinion), showing that it can be a useful tool to discriminate among children with different levels of respiratory symptom control. In addition, TRACK scores had strong correlation with medical opinion (VAS) on symptom control and moderate correlation with family opinion.

The Portuguese version of the TRACK questionnaire demonstrated good reproducibility in clinically stable children and good responsiveness in clinically unstable children. Our findings are in agreement with others that have previously found good psychometric characteristics in the original version of the questionnaire (English) [8, 9] and in Spanish and Arabic versions [10–12].

When several possible cut-off points to discriminate between children with and without respiratory symptom control were compared, the 80 points cut-off had higher area under the ROC curve (as compared to GINA criteria of symptom control) and exhibited the best balance of sensitivity and specificity. This cut-off point has been consistently identified as the most appropriate, both in the English [8] and in the Arabic [11] versions of the questionnaire.

The minimal clinical important difference (MCID) of a control tool is an important characteristic to help physicians to interpret relevant changes. The original TRACK study found that 10 or more points represent individual clinically meaningful change in respiratory control status [9]. Using the same methodology, we have found quite similar results. Distribution-based approach showed very close results (10.7 to 12.4 points), while anchor-based approach showed slighted higher results (9.3 to 24.6 points). Even so, our data can support the previously established MCID.

It is important to highlight that the group of children included in our study was somehow different from other studies performed with the TRACK questionnaire, showing more severe morbidity due to respiratory symptoms (i.e., 33% with emergency visits in the last month and 50% with wheezing episodes in the last month). All participants attended tertiary outpatient clinics where more severe cases are usually referred to.

Portuguese is one of the most widely spoken languages in the world. It ranks seventh according to some estimates [14], and it is the official language of Brazil, Portugal, a few countries in Africa (Angola, Mozambique, Guinea-Bissau) and countries/regions in Asia (Timor-Leste, Macau, Goa). A validated instrument in Portuguese may contribute to the management of respiratory diseases in pre-school children in those geographic regions.

## Conclusions

In summary, our results demonstrate that the Portuguese version of the TRACK questionnaire is an adequate tool to evaluate and follow-up respiratory symptom control in children under 5 years of age.

## Abbreviations

ACQ: Asthma Control Questionnaire
ACT: Asthma Control Test
AUC: Area under the curve
C-ACT: Childhood Asthma Control Test
CI: Confident interval
GINA: Global Initiative for Asthma
IQR: Interquartile range
MCID: Minimal clinically important difference
ROC: Receiver operating characteristic
SD: Standard deviation
SEM: Standard error of the mean
TRACK: Test for Respiratory and Asthma Control in Kids
VAS: Visual analogue scale
